# Medication Sales and Syndromic Surveillance, France

**DOI:** 10.3201/eid1203.050573

**Published:** 2006-03

**Authors:** Elisabeta Vergu, Rebecca F. Grais, Hélène Sarter, Jean-Paul Fagot, Bruno Lambert, Alain-Jacques Valleron, Antoine Flahault

**Affiliations:** * Institut National de la Santé et de la Recherche Medicale Unité, Paris, France;; † Université Pierre et Marie Curie, Paris, France;; ‡ Institut National de la Recherche Agronomique MIA, Jouy-en-Josas, France;; § IMS FRANCE, Puteaux, France;; ¶ Hôpital Saint-Antoine, Paris, France;; # Hôpital Tenon, Paris, France

**Keywords:** public health surveillance, influenza, drug sales, forecasting model, sentinel surveillance, research

## Abstract

Although syndromic surveillance systems using nonclinical data have been implemented in the United States, the approach has yet to be tested in France. We present the results of the first model based on drug sales that detects the onset of influenza season and forecasts its trend. Using weekly lagged sales of a selected set of medications, we forecast influenzalike illness (ILI) incidence at the national and regional level for 3 epidemic seasons (2000-01, 2001-02, and 2002-03) and validate the model with real-time updating on the fourth (2003-04). For national forecasts 1–3 weeks ahead, the correlation between observed ILI incidence and forecast was 0.85–0.96, an improvement over the current surveillance method in France. Our findings indicate that drug sales are a useful additional tool to syndromic surveillance, a complementary and independent source of information, and a potential improvement for early warning systems for both epidemic and pandemic planning.

## Abstract 2

Real-time over-the-counter drug sales provide an additional tool for disease surveillance.

Disease surveillance provides essential information for control and response planning. It helps identify changes in incidence and affected groups, thereby providing valuable additional time for public health interventions. Syndromic surveillance aims to use health and health-related data that precede diagnosis or confirmation to identify possible outbreaks, mobilize a rapid response, and thus reduce illness and deaths. This approach is increasingly being explored by public health officials to detect any emerging event (e.g., bioterrorist attacks) and for routine surveillance (*1**–**6*).

In France, an existing Web-based surveillance system that uses a syndromic approach by collecting weekly office visits to general practitioners provides forecasts of influenza. This approach, based on the method of analogs, produces reasonably sensitive forecasts of annual influenza epidemics (interpandemic influenza) (*7*). However, the method uses past observed patterns of influenzalike-illness (ILI) to forecast future incidence of influenza and may not be able to detect new or unusual public health events, such as the emergence of a pandemic strain of influenza or a bioterrorist attack. For this reason, we investigated other potential data sources associated with ILI that do not rely on past information to forecast incidence and are flexible enough to detect unusual increases in incidence. Here, we evaluate the potential benefit of using a complementary and independent dataset to forecast ILI and eventually to detect influenza epidemics in France. We also compare 2 surveillance methods that use a syndromic approach (one that monitors syndromes defined in clinical terms [ILI] and the other that concerns syndromes defined by using a constellation of drug-specific pharmacy sales indicators). Drug sales have the advantages of providing data on widely used products and of being available in real time. Purchases of drugs could be rapidly relayed to public health authorities, potentially providing lead time for epidemic response planning (*8*).

## Materials and Methods

### Drug Sales

We used 2 data sources aggregated at the national and regional level. The first database consists of most weekly prescription and over-the-counter (OTC) drug sales, from July 1, 2000, to August 22, 2004, provided by IMS France (http://www.imshealth.com). These data are available in quasi–real time; 7–10 days of lag time are needed for quality control and consolidation. The database includes 11,000 pharmacies throughout France (≈50% of all pharmacies) at the regional level (21 regions). The data, consisting of nearly 500 classes of medications, give the number of units dispensed or sold during a certain week for each class of drugs, identified by their codes in the European Pharmaceutical Marketing Research Association Anatomical Therapeutic Chemical (ATC4) classification. In this international classification, drugs are identified by a unique ATC4 code, which corresponds to their primary use. A panel of experts from the World Health Organization Collaborating Center selected 19 classes of medications (Table 1) likely to be prescribed or purchased for ILI. This preselection also avoids the construction of saturated models.

**Table 1:** Classes of drugs likely to be prescribed or purchased for influenzalike illnesses

Medication
Cephalosporin*†
Cough suppressant with another medication*†
Cough suppressant with bronchial-pulmonary antimicrobial drug*†
Expectorant*†
Topical nose cream*†
General rhinosinusitis preparation*
Macrolide*
Nasal decongestant*
Nonnarcotic analgesic*
Other antimicrobial agent*
Penicillin*
Rhinocorticoids without antiinflammatory agent*
Tetracycline in association with another medication*
Vitamin C only*
Pharyngeal antiinflammatory decongestant
Antiviral except anti-HIV
Cough suppressant only
Nasal antiinflammatory except corticoids
Vitamin C in association with another medication

*Significant (p<0.05) medication classes that were included in the 1-, 2-, and 3-week-ahead national predictive models. †Medication classes included in >50% of regional predictive models.

**Figure 1:**
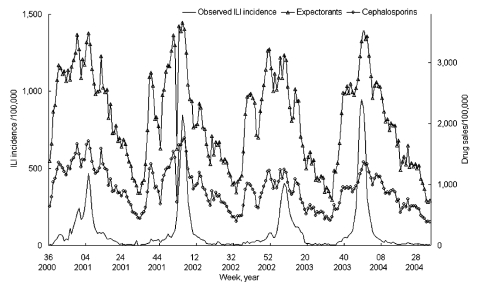
Example of weekly consumption of expectorants and cephalosporins (provided by IMS France) in phase with weekly incidence of influenzalike illness (ILI) (data from French Sentinel Network) per 100,000 population.

For all years (2001–2004), an aberration in the data for the first week of January was present, likely due to the New Year's holiday. We used the preceding and following weeks to estimate for the week of January 1. Figure 1 shows the temporal trends of sales of 2 of the 19 classes of medications (cephalosporin and expectorants) used in this analysis as an example, as well as the concomitant national ILI incidence. The other classes of drugs included in the analysis follow similar temporal trends (data available on request from the authors).

### ILI Incidence

Data on ILI incidence were obtained from the French Sentinel Network (FSN), which comprises voluntary sentinel general practitioners who update a Web-accessible database with information on communicable diseases including ILI. Weekly national and regional ongoing ILI incidence estimates are published on the Web (http://www.sentiweb.org). ILI is defined by sudden onset of fever of >39°C, respiratory symptoms, and myalgia. Epidemic weeks are defined according to a periodic seasonal regression model (based on the concept of excess deaths—here, excess illness—introduced by Serfling [9]) that is used routinely in FSN (*10**,**11*). Epidemic onset is defined as the first week in which the national ILI incidence exceeds a baseline nonepidemic threshold given by the upper limit of the 95% confidence interval of the Serfling model, provided the incidence remains above this threshold for at least 2 consecutive weeks.

### Model Construction

We used a Poisson regression model to forecast incidence of ILI based on medication sales. The model allows for overdispersion (when the variance may be larger than the mean in the raw data on ILI incidence and medication sales). The exponential of the estimated Poisson regression coefficients indicates the relative influence of each medication on the incidence of ILI in France. For the explanatory variables (i.e., drug sales data), various time lags were tested (from 0 to 4 weeks). To avoid correlation generated by several lagged values of the same variable (which can bias estimated variance of calculated coefficients), only 1 lagged version of a given explanatory variable was kept. We kept only the time-lagged variable (i.e., sales from 0, 1, 2, 3, or 4 weeks lagged) most correlated to ILI incidence.

Variables were introduced in the model by stepwise selection at the 5% significance level. Sine and cosine terms were included in the model to control for the annual seasonality of ILI incidence. Autoregressive terms (i.e., past terms of the ILI time series) were included if necessary in the final model to eliminate the autocorrelation of the residuals, a common problem in time-series data. The final structure of the model is:

Observed incidence of ILI [week *t*] = exp{intercept + *coeff* × observed incidence of ILI [week (*t* – *tILI*)] + *coeff* × sales of drug A[week (*t* – *tA*)] + *coeff* × sales of drug B [week (*t* – *tB*)] + … + *coeff* × sine(2π*t* / 52) + coeff × cosine(2π*t* / 52)},

where drug A, drug B, and the like correspond to classes of drugs marked by an asterisk in Table 1, *coeff* denotes respective coefficients of included variables, and *tILI*, *tA*, *tB*, and the like represent respective time-lags of these variables. We constructed 1-, 2- and 3-week-ahead predictive models at national and regional levels on a training dataset corresponding to the period from July 1, 2000, to September 14, 2003, when 3 outbreaks occurred. More details on the predictive models are provided in the Appendix.

### Model Evaluation

A jackknife-based resampling procedure (*12*), which produces error bounds on the estimate of regression coefficients computed from samples that leave out 1 observation at a time, was used to check the model fit. The models were validated by forecasting the 2003–04 influenza season (September 15, 2003–August 22, 2004). Models' parameters were reestimated each week with updated data on medication sales and ILI incidence. The predictions of ILI incidence from drug sales were evaluated by Spearman correlation coefficients. The correlation between observed and forecasted incidences was assessed for each forecasting horizon (1, 2, and 3 weeks ahead) for the entire 2003–04 influenza season (September 15, 2003–August 22, 2004) and for the preepidemic and epidemic weeks (October 6, 2003–January 4, 2004). When regional models were evaluated, the correlation was calculated as the average of the 21 regional correlation coefficients.

We compared the results of the proposed method to the current forecasting approach, the method of analogs, for the national model. The method of analogs, currently employed by FSN, uses weighted sums of vectors selected from historical influenza time series that match current activity to construct forecasted incidences (*7*). All statistical procedures were generated with SAS software, version 8 (SAS Institute, Cary, NC, USA).

## Results

### National ILI Incidence Forecast

**Figure 2:**
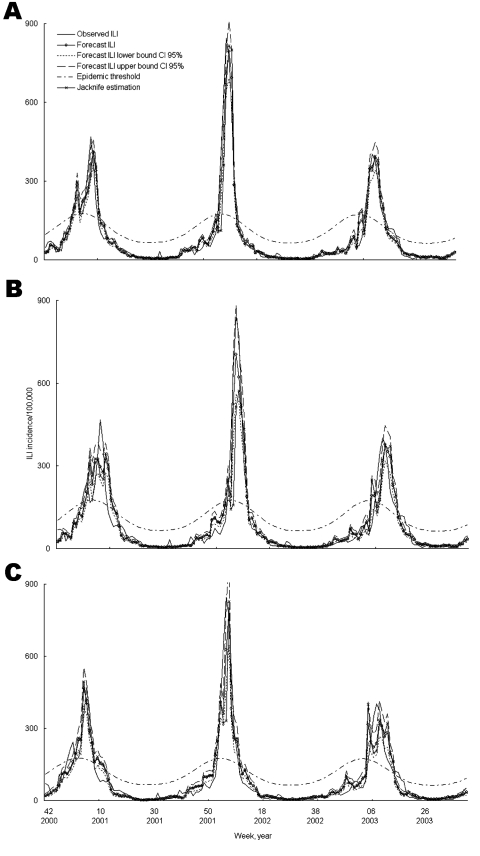
Model construction for national influenzalike illness (ILI) incidence forecasting 1, 2, and 3 weeks ahead for the training dataset (week 36 of year 2000 to week 38 of year 2003) by using a jackknife reestimation procedure (95% confidence intervals [CI] for estimations are given). Forecasted incidence is per 100,000.

The fitted predictive models for the training dataset for 1, 2, and 3 weeks ahead included 14 of the 19 preselected classes of drugs likely to be prescribed or purchased for ILI (Table 1). The correlation coefficients calculated on the training dataset between observed and model-recalculated ILI incidences were 0.94, 0.92, and 0.91 for 1-, 2-, and 3-week-ahead predictions, respectively (p<0.001, Figure 2).

The validation of these models, evaluated first on the entire period from September 15, 2003, to August 22, 2004, and secondly on the preepidemic and epidemic weeks of the 2003–04 influenza season, provided correlation coefficients of 0.85 to 0.96 (Table 2). The correlation decreased as the time horizon for the forecast increased. The method detects well the beginning of the epidemic but overestimates the epidemic size (data not shown).

**Table 2:** Forecast accuracy*†

Forecast method of ILI incidence	Forecast horizon (wks)	National level		Regional level	
		Weeks 2003(40)–2004(34)	Weeks 2003(41)–2004(01) (preepidemic and epidemic)	Weeks 2003(40)–2004(34)	Weeks 2003(41)–2004(01) (preepidemic and epidemic)
Using drug-sales data	1	0.93	0.96	0.70	0.64
	2	0.93	0.95	0.70	0.56
	3	0.91	0.85	0.69	0.54
Current method used by FSN	1	0.90	0.96	0.73	0.67
	2	0.93	0.88	0.68	0.53
	3	0.91	0.74	0.65	0.40

*Defined as the correlation coefficient between observed and predicted influenzalike incidences and calculated on the validation dataset. Correlations were computing for 1-, 2-, and 3-week-ahead prediction obtained from medication sales for 2 periods (p<0.001). Forecast accuracy was compared with that of the method of analogs. At the regional level, the value in the table was obtained by averaging the correlation coefficient over the 21 regions of France. The number in parentheses after a year refers to that week of that year. †ILI, influenzalike illness; FSN, French Sentinel Network.

The prediction accuracy of our drug sales–based model at a national level was compared with that of the current forecasting method (the method of analogs). As illustrated in Table 2, although the correlation coefficients lie in the same range of values for both methods, they are generally higher with our method.

### Regional ILI Incidence Forecast

At the regional level, 5 classes of medications appeared in at least half of the final selected models (Table 1). These variables are also the most informative in the national model (likelihood test).

**Figure 3:**
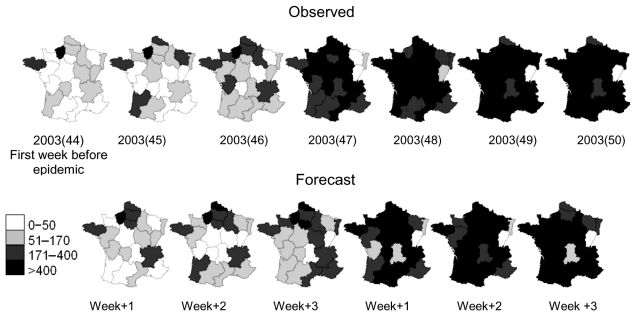
Evolution of regional influenzalike illness (ILI) incidence during the 2003–04 epidemic. The observed maps (first line) were constructed by using data from the French Sentinel Network. The forecast maps (for the first 6 epidemic weeks) 1, 2, and 3 weeks ahead show the results of the regional models when medication sales are used. The forecast horizon is indicated below each map. For example, for 2003(49), ILI predicted incidence is calculated by employing the model with data until week 47 of year 2003. Thus, the time forecasting horizon is 2 weeks.

The prediction accuracy, defined here as the average correlation coefficient for the 21 regions of France, was 0.54–0.70; it decreased slightly with the forecasting horizon. Compared to the national model, the regional models performed less well. Our regional predictive models gave higher correlation values than the method of analogs for both periods and all forecast horizons except when the ILI incidence was calculated 1 week in advance (Table 2). Forecasted versus observed regional ILI incidences were mapped (Figure 3) for the 6 first weeks of the 2003-04 influenza epidemic (November 3, 2003–December 14, 2003). Each map of predicted regional ILI incidences was constructed at 1-, 2-, or 3- week horizons. For example, for week 49 of 2003, hereafter designated 2003(49), we provided a 2-week-ahead prediction of ILI incidence, calculated by employing the model using data until the week 2003(47).

## Discussion

Our work presents a real-time approach to detect influenza outbreaks and predict trends of ILI incidence 1, 2, or 3 weeks ahead with good reliability. Our method, based on drug sales data, provides similar results as the method that uses report of visits for ILI from sentinel physicians.

The set of drug classes proposed for inclusion in the model was preselected by a panel of experts at the World Health Organization Collaborating Center, based on what they determined to be clinically relevant. Because >500 medication classes are included in the database, we selected a smaller number to avoid overparameterization. This a priori selection may have influenced our results, in particular application of the model at the regional level. Regional demographic, climatologic, and cultural differences may influence the types of medication prescribed and purchased.

After the stepwise procedure for inclusion in the Poisson regression models, the selected medication groups were both OTC and prescription medications purchased and prescribed for varying degrees of severity of ILI symptoms or complications of influenza. For example, an OTC drug such as vitamin C may be purchased before or at the onset of ILI symptoms since popular beliefs and advertising suggest it may prevent infection or lessen symptom severity (*13*), even in the lack of any evidence or regulatory approval. Cephalosporins, second-line antimicrobial agents, are often used to treat acute bacterial rhinosinusitis, a complication of ILI symptoms present for an extended period (*14*).

At the national level, our forecasting model showed overall good agreement with the observed data on ILI incidence from the FSN surveillance system (Table 2). Over time, the correlation coefficients between observed and forecast ILI time series decreased, although they remained >0.85. The fact that the model was updated on a weekly basis contributed to the overall stability of the method's accuracy. However, the method does not perform as well when used as a tool to quantify the overall epidemic impact (data not shown). Because the main objective of the method is to provide advance warning for onset of the epidemic, this limitation is less important.

At the regional level, the medications included varied from area to area, but 5 drug therapeutic classes were selected for all models (Table 1). This variation may be explained by the fact that while regional similarities exist, different external factors could influence medication consumption. In terms of the forecast accuracy, the correlation coefficients averaging over the 21 regions of France were weaker than those obtained at the national level on the validation dataset (range 0.54–0.70). This may be due to the method itself, which may perform less efficiently at a regional level, or to the quality of the observed regional ILI datasets, since they are obtained from a sample of sentinel physicians. However, the accuracy at the regional level may be sufficient for operational purposes, since the qualitative trend is more relevant than the quantitative evaluation. Using the method at the regional level also provides an additional means to follow the spatial diffusion of the epidemic wave on the basis of a robust and powerful sample of pharmacies distributed all over the country.

We compared our drug sales–based forecasting models to a nonparametric method routinely used by FSN (method of analogs) (*7*). The results obtained appear to be better than those obtained with the method of analogs, but the comparison between the 2 methods is only partial: the method of analogs exploits historical trends to forecast forthcoming ILI events, whereas the regression analysis does not (except for the autoregressive term). In the event of an influenza pandemic or other event not previously observed, our method would be more likely to predict trends that have never been seen in the recent interpandemic past than the method of analogs, which uses a 20-year time series to forecast the future.

As with all forecasting models, the results of this research highlight changes in trends rather than prediction of actual incidence. Only 4 epidemic seasons of data were available to both fit and validate the model. The addition of years of retrospective data would probably slightly improve the forecast accuracy at the national level but might greatly improve precision at the regional level.

### Value of Additional ILI Surveillance System

Our findings confirm previous studies that demonstrate the utility of using drug sales, and the timing of drug sales, compared to other indicators (*8**,**15*), as a proxy indicator of ILI activity. Several arguments support the need to consider syndromic surveillance based on drug-sales data. First, the nonspecific prodrome phase of many diseases may be self-treated before persons see a health practitioner and may therefore be more easily detected by using drug sales than laboratory surveillance or health center discharges. Second, rapidly extending the use of this method may be more feasible than creating or expanding sentinel networks of general practitioners. Drug sales are usually available in many developed countries, whereas electronic real-time surveillance of influenza or ILI is still seldom set up in most parts of the world. Third, using several sources of data with different methodologic approaches for syndromic surveillance may improve detection and prediction of trends of ILI outbreaks caused by influenza or other emerging agents. Drug-sales series represent an independent source of information, as well as reports from laboratories, general practitioners, hospitals, and death certificates, which have proved their usefulness in monitoring and assessing the impact of influenza epidemics.

### Accuracy of Drug Sales–based Surveillance System

Methods for assessing the quality of a syndromic surveillance system have been recently proposed by Buckeridge et al. (*16*). Our drug-sales time series was too short to allow a precise assessment of system's capacity to detect outbreaks appropriately. Our findings do indicate, however, that drug sales are good predictors of ILI activity recorded by the sentinel system. FSN has monitored ILI activity in France with the same method since 1984. For 21 years, during each winter, an influenza epidemic has been detected by the 2 French national influenza centers (based in Lyon and Paris) on the basis of virus isolation and simultaneously by FSN. Thus, FSN has shown a high sensitivity to detect national influenza epidemics, and we may assume that the system based on drug sales will be at least as sensitive as that of FSN. A potential advantage of the medication sales data is that their broad scope may enhance the sensitivity of detection, especially at a local level. This hypothesis has to be further assessed by evaluating a longer time period or by using simulated data for evaluation. Although using drug sales as a monitoring tool has clear benefits, detecting a nonspecific signal from our system would require further confirmation and identification of the causes of this unusual increase.

## Conclusions

Our results confirm that drug-sales data could be used as an independent additional source of information to warn of ILI outbreaks early in countries where influenza is already monitored. Drugs-sales data may be the only monitoring ILI system in countries without existing surveillance systems. The proposed method has the advantage of being both practical and relatively simple to implement. Therefore, this approach could be easily extended to other infectious diseases. In many industrialized countries, similar databases of medication sales are available in real or near-real time.

## Appendix

Weekly ILI incidences were crossed with weekly drug sales using a Poisson regression allowing for over-dispersion, in order to build 1-, 2- and 3-weeks-ahead predictive models at national and regional level. The models, with the general structure represented by the equation below, include variables selected by stepwise selection at the 5% significance level:

_
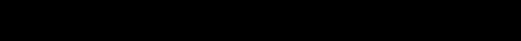
_.

*ILI_t_* is the observed ILI incidence at week *t* and _
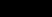
_represents the sold units of drug class *i* at week *t-t_i_*. For each class of medication, only the most correlated lagged value to the ILI incidence was introduced, in order to avoid colinearity. The terms 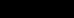
and 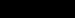 were included in the model to control for the annual periodicity of ILI and the autoregressive term (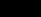) was included if necessary after the examination of the residuals for autocorrelation. For each variable, the time lags (*t_ILI_ and t_i_*) tested vary between 0 and 4 weeks.
